# Biosecurity perceptions among Ontario horse owners during the COVID‐19 pandemic

**DOI:** 10.1111/evj.14115

**Published:** 2024-06-27

**Authors:** Juliet A. Germann, Terri L. O'Sullivan, Amy L. Greer, Kelsey L. Spence

**Affiliations:** ^1^ Department of Population Medicine, Ontario Veterinary College University of Guelph Guelph Ontario Canada

**Keywords:** biosecurity, equine facilities, equine health, horse owners, qualitative research, thematic analysis

## Abstract

**Background:**

Disease outbreaks present a significant challenge to horse health and welfare and the economic stability of horse industries internationally. This is a particular concern in Ontario, Canada, where there have been frequent outbreaks of respiratory infectious diseases among horses. Despite these risks, there has been limited research on whether Ontario horse owners engage in biosecurity measures sufficient to mitigate risk of equine diseases, and whether current events such as the COVID‐19 pandemic influence attitudes towards equine biosecurity practices.

**Objective:**

To explore Ontario horse owners' perceptions, attitudes and experiences relating to on‐farm biosecurity during the COVID‐19 pandemic.

**Study design:**

Qualitative study using virtual semi‐structured interviews.

**Methods:**

Participants (horse owners, frequent horse riders and part boarders) were recruited using social media snowball sampling where advertisements were shared by equine and veterinary organisations. Interviews were conducted virtually between June and September 2022 and were analysed using reflexive thematic analysis.

**Results:**

Three key themes relating to biosecurity perceptions among the 14 participants were identified. Participants relied on minimal preventative measures (such as vaccines) where perceived risk of disease was low, but implemented additional measures including quarantine and handwashing when perceived risk of disease was high. Participants' choice of biosecurity practices often mirrored those recommended by the barn manager. Moreover, participants felt that responsibility for biosecurity was not shared equally across horse owners, with more emphasis placed on those engaging in high‐risk situations for disease spread. Despite experiencing biosecurity during the COVID‐19 pandemic, horse owners were not consistently applying these practices to their horse care routines.

**Main limitations:**

The perspectives reported here are from a small sample of horse owners and may not be generalisable to all populations.

**Conclusions:**

Our findings indicate that horse owners need improved access to and engagement with educational initiatives that emphasise the importance and purpose of all biosecurity measures.

## Summary

**Hintergrund:**

Krankheitsausbrüche stellen eine große Herausforderung für die Gesundheit und das Wohlergehen von Pferden sowie für die wirtschaftliche Stabilität der internationalen Pferdewirtschaft dar. Dies ist ein besonderes Problem in Ontario, Kanada, wo es häufig zu Ausbrüchen von Infektionskrankheiten der Atemwege bei Pferden gekommen ist. Trotz dieser Risiken gibt es nur wenige Untersuchungen darüber, ob Pferdebesitzer in Ontario ausreichende Biosicherheitsmaßnahmen ergreifen, um das Risiko von Pferdekrankheiten zu mindern, und ob aktuelle Ereignisse wie die COVID‐19‐Pandemie die Einstellung zu Biosicherheitspraktiken bei Pferden beeinflussen.

**Ziele:**

Untersuchung der Wahrnehmungen, Einstellungen und Erfahrungen von Pferdebesitzern in Ontario in Bezug auf die Biosicherheit in landwirtschaftlichen Betrieben während der COVID‐19‐Pandemie.

**Studiendesign:**

Qualitative Studie mit virtuellen halbstrukturierten Interviews.

**Methoden:**

Die Teilnehmer (Pferdebesitzer, regelmäßige Reiter und Teilpächter) wurden über soziale Medien rekrutiert, in denen Anzeigen von Pferde‐ und Tierarztorganisationen geteilt wurden. Die Interviews wurden zwischen Juni und September 2022 virtuell durchgeführt und mit Hilfe einer reflexiven thematischen Analyse ausgewertet.

**Ergebnisse:**

Unter den 14 Teilnehmern wurden drei Hauptthemen in Bezug auf die Wahrnehmung der Biosicherheit ermittelt. Die Teilnehmer verließen sich auf minimale Präventivmaßnahmen (z. B. Impfungen), wenn das Risiko einer Erkrankung als gering eingeschätzt wurde, führten jedoch zusätzliche Maßnahmen wie Quarantäne und Händewaschen ein, wenn das Risiko einer Erkrankung als hoch eingeschätzt wurde. Die von den Teilnehmern gewählten Biosicherheitspraktiken entsprachen häufig den Empfehlungen des Stallbetreibers. Darüber hinaus waren die Teilnehmer der Ansicht, dass die Verantwortung für die Biosicherheit nicht gleichmäßig auf alle Pferdebesitzer verteilt ist, wobei der Schwerpunkt eher auf denjenigen liegt, die in Situationen mit hohem Risiko für die Verbreitung von Krankheiten involviert sind. Trotz der Erfahrungen mit der Biosicherheit während der COVID‐19‐Pandemie wandten die Pferdebesitzer diese Praktiken bei der Pflege ihrer Pferde nicht konsequent an.

**Hauptlimitationen:**

Die hier berichteten Perspektiven stammen von einer kleinen Stichprobe von Pferdebesitzern und sind möglicherweise nicht auf alle Bevölkerungsgruppen übertragbar.

**Schlussfolgerungen:**

Unsere Ergebnisse zeigen, dass Pferdebesitzer einen besseren Zugang zu und eine stärkere Beteiligung an Aufklärungsinitiativen benötigen, die die Bedeutung und den Zweck aller Biosicherheitsmaßnahmen hervorheben.

## 1 INTRODUCTION

Equine infectious diseases pose a significant global challenge, as outbreaks in one region have the potential to affect other nations. The Canadian horse industry, a multi‐billion‐dollar industry generating thousands of jobs, faces concerns about equine infectious diseases due to frequent horse movement due to buying and selling horses, attending competitions and boarding.^1^, ^2^ Diseases not only pose a significant threat to equine health and welfare but can also impact the economic stability of the horse industry.^2^ There have been several outbreaks of equine infectious diseases in Ontario, Canada, including outbreaks of equine herpesvirus 1 (EHV‐1), equine herpesvirus myeloencephalopathy (EHM) and *Streptococcus equi* (Strangles).^3^ With approximately 26 490 horse facilities in Ontario, preventing and controlling diseases is crucial to minimise large‐scale outbreaks.^4^ Biosecurity encompasses a range of disease prevention and control measures designed to reduce the risk of pathogen spread.^5^ In Canada, there are no mandatory biosecurity measures for horses kept at stabling facilities; however, certain horse facilities (e.g., competition venues) may require additional measures to host equestrian shows or events. Voluntary recommendations for biosecurity measures at stabling facilities are provided by the Canadian Food Inspection Agency (CFIA) and Equestrian Canada (the governing body of equestrian sport) in the National Farm and Facility Level Biosecurity Standard for the Equine Sector.^6^ However, limited research exists regarding biosecurity adoption among Canadian horse owners, leaving a significant knowledge gap on whether the measures recommended in this standard are applied across the industry. International evidence suggests that many horse owners do not consistently implement biosecurity measures.^7^, ^8^, ^9^ A study conducted in the United States revealed that 80% of horse owners were unaware or did not have the biosecurity practices implemented at the facility where their horse was stabled.^9^ Additionally, previous research in Australia and New Zealand indicated that horse owners tend to adopt biosecurity measures only when they perceive high risk of disease threats, consistent with trends among livestock farmers.^10^, ^11^, ^12^, ^13^, ^14^, ^15^, ^16^, ^17^, ^18^, ^19^

The Health Belief Model (HBM) and Theory of Planned Behaviour (TPB) are theoretical models of health behaviour that are often used to explain behaviour change, sometimes in the context of biosecurity in various agricultural industries.^17^, ^18^, ^19^, ^20^, ^21^ These models outline how factors including attitudes, norms and perceived control can influence behaviour, but external events like the COVID‐19 pandemic may also influence these constructs. The COVID‐19 pandemic, commencing in March 2020, significantly disrupted the operation of Ontario equine facilities, leading to business closures and physical distancing measures.^1^, ^22^ As equine activities resumed, public health guidelines, including mask‐wearing and physical distancing, were enforced to mitigate COVID‐19 spread.^22^ Equestrian Canada also recommended measures including crowd limits, enhanced disinfection and improved ventilation, aligning with infection prevention and control principals akin to those recommended by the national biosecurity standard.^22^ Since these measures follow the same principles that govern equine biosecurity, there is a potential that the circumstances of the COVID‐19 pandemic influenced biosecurity attitudes among horse owners. Therefore, the objectives of this study were to describe Ontario horse owners' biosecurity perceptions and attitudes and explore whether their experiences during the COVID‐19 pandemic influenced biosecurity decision‐making and implementation.

## 2 MATERIALS AND METHODS

### 2.1 Participants

Horse owners/leasers, frequent horse riders, part boarders and barn managers/owners were eligible to participate in the study if they were aged 18 years or older and resided in Ontario at the time of the study. Part boarders are individuals who share a part of the cost of the horse with the owners and may actively participate in the regular care and riding of the horse. In this study, the part boarders involved were actively engaged in both the regular care and financial responsibilities associated with their shared horse. Participants were recruited using social media snowball sampling where veterinary and equine organisations posted recruitment posters which were further shared by individuals. Recruitment posters were also shared to smaller equine organisations/communities from the personal Facebook pages of members of the research team. Horse owners were primarily convenience sampled. However, the characteristics of horse owners were iteratively assessed to ensure a diverse representation, encompassing various types of owners.

Interested horse owners were asked to contact the first author (JG) via email to receive further information about the study. An interview date was scheduled after participants provided written informed consent and agreed to participate. Prior to the start of the interview, consent was verbally confirmed, and participants were given a chance to ask any questions. Participant recruitment continued until information power for the sample was considered adequate.^23^ Information power is a concept used to guide sampling in qualitative research and states that when a sample holds higher information power, a smaller sample size is needed.^23^ Determining when to stop collecting participants involves a stepwise process of assessing five items: broadness of the study aim, sample specificity, level of theoretical establishment, the quality of dialogue and method of analysis.^23^ The items were iteratively assessed during data collection and analysis and information power was deemed to be reached at a sample size of 14. Finalising this sample size involved a final appraisal from the research team.

### 2.2 Interviews

All interviews were conducted by the first author and took place virtually over Zoom between June and September 2022. The interviewer was a Master of Science (MSc) student with training in epidemiology and qualitative methods. They did not have an extensive background with horses, and this information was withheld from the participants so that it did not steer their responses away from their true beliefs. An interview guide containing open‐ended questions was developed to guide discussion on experiences with horse care during the COVID‐19 pandemic, perceptions and attitudes towards biosecurity, and the participant's general knowledge of biosecurity (File S1). Participants were also asked follow‐up questions based on their answers to develop depth to answers and further elaborate on their responses. Prior to the start of interviews, the interview guide was piloted with three horse owners to check for clarity, quality and understanding of questions. All interviews were audio and video recorded and transcribed verbatim using the live transcription service in Zoom. Transcripts were reviewed for accuracy by the first author and any necessary corrections were made.

### 2.3 Analysis

Interviews were analysed using reflexive thematic analysis as described by Braun and Clarke.^24^ Reflexive thematic analysis is used to identify themes or patterns across data while acknowledging the perspectives and involvement of the research team during the process. As someone with no prior experience with horses, JG approached the analysis with limited pre‐existing biases about biosecurity and horse ownership. Therefore, the analysis was based solely on what was interpreted from the interviews and through a wider literature search. The remainder of the research team had extensive experience with equine biosecurity, which allowed for a broader perspective that contributed to final theme development. The analysis was conducted from a social constructionist standpoint in which there is the belief that everyone's truth is developed through social interaction and circulation of knowledge.^25^

After ensuring accuracy of the interview transcripts, the first author listened to the audio from the interviews and read through the transcripts several times to gain familiarity with the data. Any preliminary thoughts and ideas that occurred during this stage were recorded and retained for analysis. Transcripts were imported into the programme NVivo (Release 1.7.1) to aid in analysis. The first author completed initial coding of the transcripts, which is the process of summarising or simplifying key words or phrases mentioned by participants. After initial coding was completed, codes were further refined by comparing and contrasting central concepts across the data. Patterns among codes were then conceptualised and linked together to develop categories, which were then further refined through iterative discussions among the research team to create themes. Final themes encompassed overarching trends observed across the interview data.

## 3 RESULTS

**FIGURE 1:**
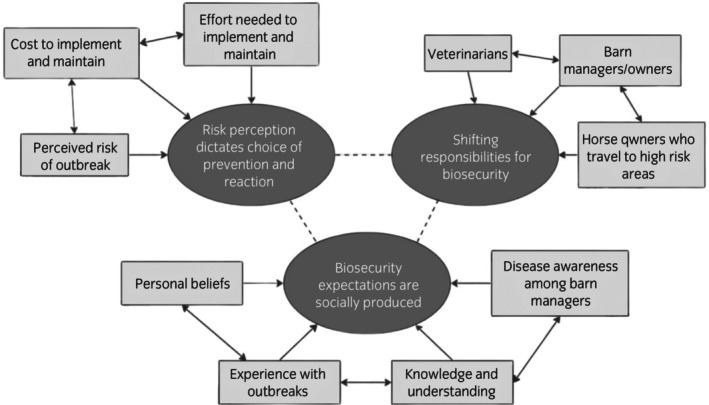
Thematic map of three themes and their corresponding influential categories constructed from semi‐structured interview data of horse owners in Ontario, Canada.

Semi‐structured interviews were conducted with 14 participants who were a mix of horse owners and part boarders. Most participants (*n* = 11) boarded their horses at a shared boarding facility, while three participants had their horses on their own property. Interview length ranged from 25 to 112 min with a median length of 33 min. Three themes were constructed from the interview data: *Risk perception dictates choice of prevention and reaction*, *Biosecurity expectations are socially produced* and *Shifting responsibilities for biosecurity*. The themes, and the influential elements within each theme, are represented in Figure 1.

### 3.1 THEME 1: Risk perception dictates choice of prevention and reaction

Participants did not always perceive the usefulness of all biosecurity measures equally, and instead categorised them as either preventative measures or reactive measures. Decisions surrounding which biosecurity measures should be used were based on whether participants felt that it was justified for their situation. Justification came from considering the perceived risk of an outbreak, the cost of the measure and the effort needed to implement and maintain the measure. While cost and effort were important considerations, perceived risk of a threat was the greatest influence on justifying whether certain biosecurity measures were needed. When risk of disease was perceived to be low, participants tended to rely on protections provided by vaccinations. Vaccines were one of the only measures that participants described were generally accepted among horse owners to frequently and properly implement:

There is kind of a general understanding that everyone gets their horses vaccinated for certain diseases. —Participant 3

However, even the use of certain vaccines was dependent on perceived risk:

Not everybody's going to vaccinate for strangles, not everybody's going to vaccinate for the things that they aren't worried about. —Participant 1

Other measures, such as quarantine, were still used as preventative measures but were more regularly used when there was a high perceived risk of disease, such as an outbreak in the local area. However, unlike vaccinations, quarantine was more often found to be improperly or ineffectively used:

I think people don't even quarantine their horses. They'll have it arrive on the farm with no testing, no knowledge of who these people are, and they'll chuck it out with the herd and just see if they all get along together. —Participant 10

Quarantine was often a long‐term process over the span of two‐weeks that required high effort and high cost to implement properly. As a result, some participants described how others simply went through the motion of quarantining without the necessitated carefulness. While participants considered quarantine an effective preventative measure, there was frustration towards other horse owners who often practiced what they termed ‘biosecurity theatre’ and were not being as careful as they should be:

They're not really thinking about all those little things, like keeping the home horses away from the quarantine horses, within 10 feet […] The other day, when [the barn staff] had the horses just come in into the quarantine area, they walked my horses literally right in front of [the other horses] right past their door. —Participant 8

Furthermore, without perceived risk of disease, some participants made exceptions to why their horse should either not quarantine at all or why they should only quarantine for a part of the designated time. Often, these exceptions were associated with the age or social level of the horses:

When we first brought our little filly, she was in a separate paddock. But she was only separated for a little while […] It was a long trip that was very exhausting for her, and she'd never been on a trailer, she's never had any of these things. So, she was just exhausted when she arrived and it was in the middle of the night… Sunday, she jumped out of the paddock when we arrived. So, after that happened, instead of, you know, putting her back in, the manager felt that putting her in with other horses at that time was a good idea. —Participant 1

Biosecurity was often perceived as direct‐to‐horse interventions, including measures such as quarantine and vaccinations, albeit with varying levels of implementation. Conversely, indirect interventions, carried out at the horse owner level, included measures such as hand washing, boot washing and changing clothes between barns. These indirect interventions were often designated as reactive measures, particularly used only during high‐risk situations:

No, we don't have any boot washing or hand washing stations at the moment. I've seen them before when strangles was really high. We did all have to clean our boots and change everything between barns. —Participant 10

This distinction between direct biosecurity (which was viewed as preventative) and indirect biosecurity (which was viewed as reactionary) further reinforced the designation of biosecurity measures to be used during different times. However, if disease were to still get through both the preventative and reactionary measures, it reinforced the belief of some participants that disease was unavoidable. Despite this, some participants were more willing to put effort into what they referred to as ‘smaller’ preventative measures rather than putting larger effort in reactionary measures:

If [horses] are going to get [diseases], it is what it is, but it's mostly the work involved in treating or managing them is a huge amount of time, so I look at small daily biosecurity measures as things I can do to kind of potentially save me time in the future. —Participant 11

Participants often used perceived risk as a benchmark to implement biosecurity. Yet, perceived risk was highly sensitive to changes in surrounding factors and could increase and decrease based on available information. Participants mentioned hearing about outbreaks via word of mouth from other horse owners, from their veterinarian, and also from social media. This indicated a subjective understanding of perceived risk which resulted in justifying the use of biosecurity measures. However, there was a concern that horse owners may not report an outbreak due to the fear of stigma, as described by one participant:

You know, it's almost like people fear the stigma that their horses have to quarantine than actually the sickness. Can't go to other farms, people don't trust you, they don't want to come to you, and if they don't want to come to you and you get some of the media, you don't get money. —Participant 10

While perceived risk of an outbreak was an important influential factor for biosecurity use, in some cases, it was not enough to motivate owners to improve their biosecurity practices. Instead, experiencing an outbreak was thought to teach both horse owners and barn managers about proper biosecurity procedure. However, this was a reactive mentality that was viewed as problematic by participants, since others were not learning about proper biosecurity until it was too late:

I've been to plenty of barns that have a horse that is diagnosed with strangles or something and they have no idea how to deal with it, and they really don't understand the importance of why we're telling them to put these biosecurity things in place. —Participant 3

Furthermore, perceived risk was also influenced by an individual's personal beliefs and how risk averse they were to disease outbreaks. Some participants believed that this could be influenced by their profession and experiences outside the barn:

My profession is in infection prevention and control in healthcare. So yeah, so I'm very knowledgeable in infectious diseases, but from a human aspect. I mean of course there's crossover. But, you know, when it comes to promotion of vaccination and safety measures in terms of infection prevention control, it's kind of up my alley. —Participant 1

When considering the impact of COVID‐19 on equine biosecurity practices, there was a low sense of relevancy between the two experiences. Due to the pandemic, public health guidelines were implemented in barns, inadvertently providing protections to both humans and horses. There were similarities between the COVID‐19 pandemic and the equine sector, where in both contexts, biosecurity was used reactively rather than proactively. However, when many of the guidelines were dropped, horse owners did not appear to learn from their experience with public health guidelines:

I guess during outbreaks, there's a sign… we're supposed to ‐ and not everybody does it ‐ but when we get to the barn, we wash our hands. And then, before we leave the barn, we're supposed to wash our hands again. It's been relaxed quite a bit more. It was mostly for COVID that it was in place. —Participant 2

While some horse owners made connections between the two experiences, if there was a disconnect between understanding the effectiveness of biosecurity and understanding disease transmission in both horses and humans, then there was also difficulty in understanding the usefulness of the practices:

I feel like with COVID, the biggest takeaway everyone took was wash your hands and sanitize surfaces, and I mean, obviously, we can implement that into a barn, but that's not quite going to fix [the spread of disease]. I mean, at the same time, the services can definitely help, but me washing my hands isn't going to stop a horse from getting a disease. —Participant 13

However, participants also recognised that the importance of some measures was being better understood by other horse owners after their experiences with COVID‐19, especially when they had to implement additional biosecurity practices. Rather than biosecurity being perceived as a ‘big deal’, horse owners were more accepting of the measures needing to be implemented:

Being able to have experience with COVID, and when it came time to explaining what to do when there was a herpes virus outbreak at the barn, [the boarders] were a lot more accepting of the change compared to if they hadn't experienced COVID or any major outbreaks of anything, then they would have probably been a lot more frustrated with the increased measures. —Participant 5

### 3.2 THEME 2: Biosecurity expectations are socially produced

Barn managers set the theme for rest of the barn not only with biosecurity implementation but also with biosecurity perceptions. Participants who had a generally good understanding and knowledge of biosecurity often credited their barn managers for being aware of proper biosecurity procedures and also for monitoring outbreaks in the surrounding area:

[The barn manager] is actually really big on biosecurity and making sure everything is protected in terms of, like, bacteria you brought in from other places. And she's really on top of keeping yourself up to date on any sort of pathogen that can be spread amongst the horse world, like EHV‐1 and strangles are two really big ones that we monitor a lot. —Participant 14

While there were many factors that went into choosing a barn to stable their horses, participants described how owners tended to gravitate towards barns that upheld shared beliefs. Participants that disagreed with barn managers on their approach to management and biosecurity practices would often try to find a better fitting barn. Some horse owners (especially novice owners) relied on their barn manager to provide them with information, which subsequently influenced what they believed was necessary for equine care. Exposure to proper procedures and good understanding of biosecurity was perceived as crucial to a horse owners/riders' development; however, there was frustration that some equestrians who were early in their career were not getting proper education from their facilities. Some participants perceived that it was the responsibility of the manager/facility to be teaching early career riders on what is appropriate:

Especially in lesson barns, you get people that… It's not their fault, they're just starting in the horse world, and it's not something that is taught in the horsemanship, or just the barn management skills. Most of the time it is not taught to the child or the adult that goes for a weekly riding lesson. They just go, they groom the horse, and then they're not taught this. So, it's something that's lacking in the education of the future riders. —Participant 2

Since there was a reliance on barn managers to set the social norms regarding what was acceptable for the barn, boarders used this to justify that the biosecurity measures they were implementing were ‘good enough’ because their barn manager said so. Owners that valued biosecurity either (1) took initiative into learning better biosecurity practices, or (2) went to a new barn where barn managers implemented additional practices. Participants described there being a range in outbreak precaution levels at facilities, which was dependent on the manager:

I've seen it like a 50/50 toss between some of the barns I go to. Some of them, yeah, are a little bit more concerned than others. Other barns don't seem to have [biosecurity] unless there's an actual outbreak going on. Other barns don't really have that same level of caution. It all depends on management and owner styles. —Participant 14

The lack of outbreaks at facilities reinforced participants' beliefs for not needing to implement proper biosecurity measures and procedures. This led to a shared complacency among boarders:

I think a lot of it's just like, we've done it this way for this long and it's never been a problem, so why do we need to change it now? Which I find is often a view of people in the horse industry. It hasn't hurt us yet, so why should we do anything about it? —Participant 3

The influence of social norms on barn communities also impacted how horse owners perceived appropriateness of certain biosecurity measures. When barn management implemented biosecurity, the norm for that facility became what was implemented. Without that influence, biosecurity may not be implemented at all:

I don't think people would be getting vaccinations and things unless the barn had a requirement, and [the barn] had a requirement that they needed to have them. —Participant 8

As a result of a lack of awareness of biosecurity in the horse industry, and a reliance on social cues, a social norm was developed where some participants worried about being ‘overly cautious’, especially if there was a low perceived risk of an outbreak:

We're not overly cautious, we're not, you know, crazy about it, but try to live at a moderate cautious level. Enough to be safe most of the time, but not limit us from actually going out and doing the things we enjoy, like going to a horse competition or going camping somewhere. —Participant 12

Participants also described the difficulty in determining whether all, or any, biosecurity measures were needed based on the lifestyle and activities of the horse. Therefore, participants looked to social cues to determine what should be used. This became problematic because if no one was implementing any biosecurity measures, then biosecurity would not be practiced, resulting in a cycle of reinforcement. Similarly, the absence of biosecurity practices created a culture of disregarding biosecurity measures:

It's kind of like a taboo subject. I didn't even really say anything when my foal was sick because he was for sale at the time, so I didn't really want it, kind of, tarnishing his sale… his saleability. However, people around me knew he was sick, knew what he was sick with, all the other barns where my other horses were. They knew and they knew my plan, and they were fine with what I was doing. —Participant 11

The impact that social pressure had on practicing sub‐par measures and maintaining them at a sub‐optimal level was perceived as not only influencing other horse owners, but also influencing veterinarians:

When I talk to some vets about it, they kind of told me that they think that their clientele would look at them like they were crazy if they showed up in coverall and boots instead of showing up in like jeans, a polo, and Blundstones. —Participant 3

However, some participants noticed a shift in what was considered ‘normal’ following the COVID‐19 pandemic. Prior to the pandemic, participants noticed that many competitors at equestrian events would share equipment, use communal drinking areas for horses, and mix with other barns. During the pandemic, competitors were required to bring their own supplies and practice physical distancing due to public health guidelines. As these requirements were relaxed, competitors continued bringing their own supplies and practicing physical distancing, even though they were no longer required:

But from what I've seen so far, the first few competitions of the year, a lot of people are just doing what they've gotten used to over the last 2 years and, kind of, do their thing and head back to their trailer, and don't mix horses and stuff all that much. —Participant 3

### 3.3 THEME 3: Shifting responsibilities for biosecurity

Participants expressed frustration over the lack of mandatory biosecurity protocols on both equine facilities and within the horse industry in general. As a result of the lack of mandatory protocols, horse owners and facilities could implement as much, or as little, biosecurity measures as they would like, aside from the requirements at Equestrian Canada sanctioned events. This variability in biosecurity implementation across the Ontario horse industry was viewed as problematic since the effort and care some horse owners put into biosecurity was perceived as diminished by those who did not. Participants perceived that other horse owners did not take accountability or responsibility for biosecurity in the horse industry. As a result, the horse owners who did put in care and effort were tasked with additional responsibilities:

When I think of somebody bringing strangles in, and they're not vaccinated, like, why am I paying hundreds and hundreds of dollars to get my 3 horses clear, so that they can even move somewhere else? And why am I having to pay for that, if you made this choice and you don't have any kind of accountability for it? —Participant 8

Participants perceived that biosecurity was primarily the responsibility of those whose horses were at higher risk of contracting and spreading disease. Responsibility for biosecurity was also placed on higher‐level figures, such as event organisers, which led to some horse owners attributing blame to these individuals for not ‘policing’ events more and barn managers for not taking responsibility over horse owners in their barn. Barns that had a poor biosecurity implementation were often considered the fault of the barn manager. Furthermore, some participants expressed the need for policing across equine facilities in high‐risk areas (i.e., competitions) and during outbreaks because there was a lack of trust that barn managers or event organisers were doing what was needed at the time to prevent spread. While certain equestrian events (such as events sanctioned by Equestrian Canada) required proof of vaccination to be able to compete, participants wanted to see more regulation for preventing the mixing of horses from different barns. In contrast, unsanctioned events did not have any regulations, which was viewed as problematic for competitors:

I try and be as careful as possible about how often she goes off property, and where we go. So, we tend to avoid schooling shows for that reason, because they don't require proof of vaccination. So, we tend to stick to silver or gold rated shows [Equestrian Canada regulated competitions] just because anytime horses gather, it's a risk. —Participant 11

There was also a desire for there to be a ‘designated expert’ available to assess the quality of biosecurity practices at equine facilities as well as monitor how outbreaks were being handled. Some participants described this being in the form of an external person to oversee how well measures were being implemented:

Just more visits to see if [barn managers] are actually working and doing everything. I know it's hard to find somebody who can come in and, like, watch and see what's going on but it would have been nice to have weekly visits to see…Oh, has there been any fevers like instead of… the only visits that happened is when we called to complain. —Participant 9

Concerns about the improper use of biosecurity measures was also directed at horse owners who were at higher risk of acquiring disease from travelling off‐property:

Now the concern comes for me when people are traveling, and showing, and going to venues where you can have exposures, and you would want to make sure that your horse, well that their horses, didn't bring anything back. —Participant 1

This was associated with a lack of awareness about how diseases spread, for example, possible spread via communal water sources and shared equipment at events:

I remember I posted something [on Facebook] about, well, ‘Don't put the hose nozzle in your bucket at horse shows’, and the amount of people that were like, ‘Oh my god, I never thought about that’. I was like, are you nuts? —Participant 11

## 4 DISCUSSION

This study provides insight into the perceptions and attitudes towards biosecurity held by a selection of Ontario horse owners. This includes the importance of perceived risk on the categorisation of biosecurity measures into prevention and reaction, the barn manager being an important influencer for social norms relating to biosecurity, and an unequal sense of responsibility and accountability for biosecurity within the horse industry. These themes suggest that existing biosecurity perceptions, and subsequent implementation of biosecurity practices, may provide challenges to optimal disease prevention and control should there be a wide‐spread outbreak.

While most vaccinations are not a required practice, they are highly recommended for all horses as they are an effective measure against certain diseases.^5^, ^26^ However, vaccinations do not provide 100% protection for all diseases and are therefore recommended to be used in combination with other biosecurity measures.^6^, ^26^ Similar to the discussion around COVID‐19 infection prevention and control practices, equine biosecurity practices can be related to Reason's Swiss Cheese Model.^27^, ^28^ The Swiss Cheese Model provides a useful analogy which, in its simplest terms, describes how biosecurity can be understood with using Swiss cheese as a metaphor for layering different biosecurity measures together to prevent pathogens from passing through the gaps in the cheese, which represent the flaws in each biosecurity measure.^28^, ^29^ Evidently, preventative biosecurity was perceived by horse owners in this study as a single measure rather than consisting of several smaller routine measures. This adverse reaction to having to implement multiple measures for long periods of time has been demonstrated during the COVID‐19 pandemic with the phenomenon known as ‘pandemic fatigue’.^30^ Pandemic fatigue has been recognised as a result of sustained behavioural change that impacts an individual's social, physical and economic environment.^30^ Similarly, reactionary biosecurity measures such as quarantine and boot washing were perceived as behavioural change procedures with high effort needed which can only be sustained for a limited time. This may explain the general acceptance of vaccinations among study participants, since vaccinations do not require sustained behavioural change and the low effort justifies its use as a primary preventative measure when there was a low perceived risk of outbreak. This finding is synonymous with a study conducted in Great Britain that explored exotic disease preparedness among equine veterinarians.^31^ Similar to the reactive perception towards biosecurity found in this study, equine veterinarians had a tendency to prioritise reactionary medicine over preventative medicine in their day‐to‐day practice. While veterinarians were aware that they should be taking proactive steps towards prevention, it was still not something that was frequently practiced. Focusing on good equine management practices, including preventative biosecurity, should be better emphasised.^32^

When deciding which measures to implement, horse owners assessed perceived risk, effort needed to implement and maintain the behaviour and the cost of the measure. This has also been encountered and explained by health behaviour models commonly applied to other agricultural industries, such as the HBM and TPB.^20^, ^21^ Both models explore how demographic and socio‐psychological characteristics influence an individual's intentions and subsequent behaviours.^17^ However, the HBM is more specific to health‐related behaviours and will be the model used for comparison herein. While individuals consider their subjective understanding of how susceptible they are to contracting disease, they will not undergo behavioural change if they do not believe the measure to be effective or feasible.^21^ At the same time, individuals also weigh social and health risks, as well as perceived barriers for implementation, such as cost and time.^21^ The HBM also discusses how certain stimuli are sometimes needed to provide the necessary push into action, such as experiencing symptoms, social influence, or discussions with a health professional.^21^ However, underlying these perceptions are demographic and socio‐psychological values that ultimately influence an individual's intention and action. Livestock farmers' behaviours towards biosecurity have also been explained by the HBM and TPB. In a study reviewing determinants of farmers' adoption of infection prevention practices, farmers considered feasibility of the practices, their own knowledge of the practice, and their level of positive perceived behavioural control.^19^ The influential factors described in this model are consistent with those identified in this study, which may help explain why horse owners might choose to practice certain behaviours over others.

In Canada, the occurrence of most equine pathogens does not need be reported at the provincial or federal levels, leading to challenges regarding biosecurity decision‐making based on perceived risk of outbreaks. Evidently, low perceived risk among participants meant that participants prioritised using only vaccinations as a preventative measure and did not further implement other biosecurity measures. The reliance on vaccinations as a singular preventative measure might also be attributed to regulations across the industry, where some venues require attending horses to be vaccinated to participate in a competition. If horse owners are uncertain about which additional measures to implement beyond vaccination, this may either positively or negatively influence biosecurity behaviour. For example, livestock producers that are uncertain about the efficacy of disease prevention strategies may hesitate to apply on‐farm measures.^19^ Uncertainty also has the ability to influence different economic and health behaviours, such as preventative care.^33^ For example, in a study about breast cancer screening, women who had a higher understanding of risk for the development of breast cancer were more likely to participate in breast cancer screening practices.^34^ In contrast, uncertainty can also prevent individuals from engaging in health behaviours. In Australia, there have been multiple Hendra virus events in both humans and horses, leading to the development of a vaccine.^35^ However, despite the recommendations by government officials, there has been slow uptake of the vaccine among horse owners.^35^ One study reported a high level of uncertainty towards the vaccine among horse owners regarding the vaccine, attributed to its potential side effects and the lack of clarity surrounding an emerging zoonotic disease.^35^ Furthermore, some horse owners considered themselves at a low risk of an outbreak and would prefer using other hygienic and biosecurity practices over the vaccine.^35^ A possible reason for this contrast could be that the vaccines used in Canada have a longer history and are supported with extensive scientific literature compared with the Hendra virus vaccine, which was developed rapidly in response to the outbreak. Zoonotic diseases like the Hendra virus have also not been reported in Canada and may cause a difference in perceptions. Furthermore, while there may be many influential factors contributing to differences in risk perceptions across countries, one explanation may be the cultural influence on risk, which states that risk is a product of a society's cultural values.^36^ These societal and cultural determinants also influence which level of risk is perceived as acceptable or unacceptable.^37^ Therefore, risk perceptions may vary significantly across societies and cultures and is not a broad perception that can be applied to all. It is also important to consider that there are strict regulations regarding vaccinations at sanctioned events, where indirect measures implemented at the horse owner level are comparatively less stringent. This dynamic may influence how horse owners perceive the importance of vaccinations in maintaining horse health.

Study participants described how their barn manager influenced biosecurity perceptions and implementation in the barn. They observed complacency among fellow horse owners, who seemed indifferent to sub‐par measures. This is similar to a phenomenon known as the ‘bystander effect’.^38^ The bystander effect, observed in various contexts, theorises that if there are multiple witnesses to an emergency, then people are less likely to intervene compared with situations where there is only one witness.^38^ While not an emergency, a possible reason for complacency of biosecurity is that other horse owners sometimes feel that others should be taking responsibility for biosecurity, especially in a group setting. In a professional workplace setting, it has been observed that employees will wait for their co‐workers to voice information to their manager before they decide to voice information themselves.^39^ This could be extrapolated to a barn setting, where boarders may hesitate to express their worries to a barn manager, especially since barn managers were considered an authority figure. Furthermore, the ‘the spiral of silence’ theory suggests that individuals feel less willing to speak out if they feel that it goes against a predominant public opinion.^40^ In the horse industry, there are many social and management norms that, in addition to peer pressure, have raised animal welfare issues.^41^, ^42^ Within this study alone, there were concerns of stigma influencing horse owners' and facilities' desire to report outbreaks. If horse owners perceive that the predominant opinion is to have sub‐optimal levels of biosecurity, then there will be social pressure to not speak out against what is or what is not being implemented at equine facilities. By normalising proper biosecurity practices in everyday use, the social norm will move away from the current norm of low biosecurity use across the industry.

The existing social norm regarding biosecurity is also linked to the perceptions of who should be responsible for biosecurity within the horse industry. Biosecurity responsibility involves taking initiative to learn about practices that can help mitigate disease depending on the horse's risk. However, since there are no mandatory biosecurity requirements for equine facilities within Canada, horse owners are placed in a position of responsibility to maintain and manage their horses' health.^43^ As this study shows, horse owners have a tendency to place responsibility on higher level figures, such as barn managers, to be overseeing the implementation of biosecurity measures rather than taking initiative for implementing their own practices. Even in situations where biosecurity was required (such as vaccination requirements at equestrian events), there were concerns that it was not being done properly since everyone in the horse industry may not value biosecurity equally. This may be caused by what is known as the self‐serving bias, where people are more likely to take responsibility for positive outcomes but place blame when there is an undesirable outcome.^44^ A possible reason for this bias is the perception of control: positive outcomes are due to an individual's personal control while undesirable outcomes are considered out of their control.^44^ This is a logical connection to what horse owners perceive: when there is no outbreak, they attribute it to their good biosecurity practices, but when there is an outbreak, it is because of other facilities' poor biosecurity practices. Historically, there has been a tendency for people to place blame for an outbreak on either a culture or an ethnic group as a whole.^45^ Placing blame on these groups are often a result of social inequalities, and the implementation of public health guidelines reinforce existing prejudices and biases.^45^ Another reason for placing responsibility on others is that it is easy and means that there is no need to take accountability for an outbreak. Horse owners with a better understanding of biosecurity felt more responsible for their actions, and as such, did not feel pressured to upkeep poor management norms. Similarly, farmers that had a higher sense of responsibility towards animals and humans were also found to have better knowledge of biosecurity.^17^, ^19^ This means that there may be a lack of overall biosecurity knowledge that is resulting in perceptions of unequal responsibility and accountability.

While this study cannot conclusively say whether biosecurity perceptions have evolved as a result of the COVID‐19 pandemic, the results suggest that horse owners did not translate their biosecurity experiences during the pandemic to horse care. However, there were similarities across the experiences, especially relating to social pressures. During the pandemic, there were polarising opinions on public health that pressured people to fall within social categories.^46^ Similarly, social pressures within the horse industry were found to affect people's decision making, including veterinarians and horse owners. During the COVID‐19 pandemic, there were difficulties with risk mitigation due to miscommunication of information on social media sites.^47^ Within the horse industry, there are also difficulties with miscommunication of information where erroneous information gets shared by word of mouth or on social media. Finally, in both contexts, there are problems with wilful ignorance where people consciously avoid finding the right information either out of spite or to avoid putting in effort.^48^ Knowing that these barriers are common difficulties in risk communication and mitigation for both animal and human outbreaks, it is vital that they are targeted in future outbreaks. This provides opportunities for incorporating One Health into biosecurity education. One Health recognises the interconnected health of humans, animals and the environment.^49^ By applying a One Health approach to biosecurity teachings, understanding of disease prevention theory and application can be more widespread across these pillars.

Finally, it is important to acknowledge the limitations in this research. As a qualitative research study, the quality of data and its potential transferability is determined by the information power of the sample.^23^, ^24^ Transferability refers to the richness present within the sample and whether it can be reasonably applied to different contexts.^24^ Readers should consider their own position and experiences within the horse industry to determine if the findings are transferable to their context. Qualitative research recognises the role of the researcher in driving the analysis process, which means that the interpretation of the data may vary from researcher to researcher.^24^ Furthermore, implicit bias is a bias that can affect both the researcher and participants due to unconscious cultural biases that influences judgement.^24^ As a researcher, implicit bias can be avoided by not stereotyping participants and assuming that they fit within the horse industry stereotype.^50^ However, as the author who conducted the analysis (JG) is a researcher with limited time within the horse industry, implicit bias may be reduced compared with researchers who have spent more time in the industry. Furthermore, there are many different groups within the horse industry which may have different practices and therefore hold perceptions different from the horse owners interviewed in this study. For example, many of these themes were within the context of boarding. Horse owners who have their own facility might find that they do not relate to this context. Furthermore, despite barn managers being considered a large influence for biosecurity perceptions, they were not interviewed in this study. Finally, the horse owners who took part in this study might also have a better general understanding of biosecurity than those who did not take part in the study, which would be a factor in the identified themes.

## 5 CONCLUSION

This study aimed to explore Ontario horse owners' experiences, attitudes and perceptions towards equine biosecurity during the COVID‐19 pandemic. There are several recommendations made to improve access and engagement with biosecurity, including: (a) emphasising the importance of preventative biosecurity; (b) normalising preventative measures within facilitates to reduce the impact of social pressures; (c) integrating One Health into biosecurity education to improve how disease prevention and control is understood across diseases and species; and (d) ensuring that data are accessible to both researchers and horse owners to help inform biosecurity decision‐making. Further research should focus on understanding perspectives of different groups within the horse industry that have been identified as being more influential to biosecurity use, such as barn managers and horse owners with no experience with disease. Recommendation for future research should also focus on gathering more information on the types of biosecurity practices used at equine facilities and how their implementation may be related to personal beliefs.

## FUNDING INFORMATION

No specific funding was received for this work. Stipend funding for Juliet A. Germann was provided by the University of Guelph.

## CONFLICT OF INTEREST STATEMENT

The authors have declared no conflicting interests.

## AUTHOR CONTRIBUTIONS

**Juliet A. Germann:** Conceptualization; investigation; writing – original draft; methodology; validation; visualization; software; formal analysis; data curation. **Terri L. O'Sullivan:** Conceptualization; investigation; validation; writing – review and editing; project administration; supervision; methodology. **Amy L. Greer:** Conceptualization; investigation; validation; writing – review and editing; project administration; supervision; methodology. **Kelsey L. Spence:** Conceptualization; investigation; validation; writing – review and editing; methodology; project administration; supervision; data curation.

## DATA INTEGRITY STATEMENT

Kelsey L. Spence had full access to all the data in the study and takes responsibility for the integrity of the data and the accuracy of data analysis.

## ETHICAL ANIMAL RESEARCH

This study was approved by the University of Guelph Research Ethics Board (REB # 22‐03‐034).

## INFORMED CONSENT

Both written and verbal participant consent was received prior to the start of the interviews.

### PEER REVIEW

The peer review history for this article is available at https://www.webofscience.com/api/gateway/wos/peer-review/10.1111/evj.14115.

## Supporting information


**Data S1:** Supporting information.

**File S1:** Interview guide.

## Data Availability

The data that support the findings of this study are available from the corresponding author upon reasonable request: Open sharing exemption granted by the editor.

## References

[1] Evans V . The State of the Industry 2010 Canadian Equine Industry Profile Study. 2011 [cited 2023 April 1]. Available from: https://www.equestrian.ca/cdn/storage/resources_v2/mzpQQ3p39NRcMys6K/original/mzpQQ3p39NRcMys6K.pdf

[2] Spence KL , O'Sullivan TL , Poljak Z , Greer AL . Estimating the potential for disease spread in horses associated with an equestrian show in Ontario, Canada using an agent‐based model. Prev Vet Med. 2018;151:2–28. 10.1016/j.prevetmed.2017.12.013 29496102

[3] Ontario Animal Health Network . Ontario equine disease alerts. 2023 [cited 2023 April 1]. Available from: https://www.oahn.ca/resources/ontario-equine-disease-alerts/

[4] Equestrian Canada . Response to COVID‐19 for Canada's active equines. [cited 2023 April 1]. Available from: https://www.equestrian.ca/cdn/storage/resources_v2/NbDcrdqcni6mPSBBv/original/NbDcrdqcni6mPSBBv.pdf

[5] Weese JS . Infection control and biosecurity in equine disease control. Equine Vet J. 2014;46:654–660. 24802183 10.1111/evj.12295 PMC7163522

[6] Government of Canada . National farm and facility level biosecurity standard for the equine sector. [cited 2023 July 15]. Available from: https://inspection.canada.ca/animal-health/terrestrial-animals/biosecurity/standards-and-principles/equine-sector/eng/1460662612042/1460662650577

[7] Crew CR , Brennan ML , Ireland JL . Implementation of biosecurity on equestrian premises: a narrative overview. Vet J. 2023;292:105950. 36642241 10.1016/j.tvjl.2023.105950

[8] Kirby AT , Train‐Dargatz JL , Hill AE , Kogan LR , Morley PS , Heird JC . Development, application, and validation of a survey for infectious disease control practices at equine boarding facilities. J Am Vet Med Assoc. 2010;237(10):1166–1172. 21073388 10.2460/javma.237.10.1166

[9] Vanderman KS , Swinker AM , Gill BE , Radhakrishna RB , Kniffen DM , Staniar WB , et al. Survey on the implementation of national equine identification in the United States. J Equine Vet. 2009;29(12):819–822.

[10] Wiethoelter AK , Sawford K , Schembri N , Taylor MR , Dhand NK , Moloney B , et al. “We've learned to live with it”—a qualitative study of Australian horse owners' attitudes, perceptions and practices in response to Hendra virus. Prev Vet Med. 2017;140:67–77. 28460752 10.1016/j.prevetmed.2017.03.003

[11] Schemann K , Taylor MR , Toribio JALML , Dhand NK . Horse owners' biosecurity practices following the first equine influenza outbreak in Australia. Prev Vet Med. 2011;102:304–314. Available from: http://sydney.edu.au/vetsci/biostat/macros/ 21893356 10.1016/j.prevetmed.2011.08.002

[12] Rogers CW , Cogger N . A cross‐sectional survey of biosecurity practices on Thoroughbred stud farms in New Zealand. N Z Vet J. 2010;58(2):64–68. 20383239 10.1080/00480169.2010.65087

[13] Duong TT , Brewer TD , Luck J , Zander KK . Understanding biosecurity threat perceptions across Vietnamese smallholder farmers in Australia. Crop Prot. 2019;117:147–155.

[14] Vermeulen L , Van Beirendonck S , Bulens A , Van Thielen J , Driessen B . The perception of biosecurity, management, and labour of batch management production systems among pig producers. Can J Anim Sci. 2017;97(4):590–598.

[15] Kristensen E , Jakobsen EB . Danish dairy farmers' perception of biosecurity. Prev Vet Med. 2011;99(2–4):122–129. 21345504 10.1016/j.prevetmed.2011.01.010

[16] Brennan ML , Christley RM . Cattle producers' perceptions of biosecurity. BMC Vet Res. 2013;9(71):1–8. 23574789 10.1186/1746-6148-9-71 PMC3626881

[17] Renault V , Damiaans B , Humblet MF , Jiménez Ruiz S , García Bocanegra I , Brennan ML , et al. Cattle farmers' perception of biosecurity measures and the main predictors of behaviour change: the first European‐wide pilot study. Transbound Emerg Dis. 2021;68(6):3305–3319. 33225630 10.1111/tbed.13935

[18] Garforth CJ , Bailey AP , Tranter RB . Farmers' attitudes to disease risk management in England: a comparative analysis of sheep and pig farmers. Prev Vet Med. 2013;110(3–4):456–466. 23490144 10.1016/j.prevetmed.2013.02.018

[19] Ritter C , Jansen J , Roche S , Kelton DF , Adams CL , Orsel K , et al. Invited review: determinants of farmers' adoption of management‐based strategies for infectious disease prevention and control. J Dairy Sci. 2017;100(5):3329–3347. 28237585 10.3168/jds.2016-11977

[20] Ajzen I . The theory of planned behavior. Organ Behav Hum Decis Process. 1991;50:179–211.

[21] Janz NK , Becker MH . The health belief model: a decade later. Health Educ Q. 1984;11(1):1–47. 6392204 10.1177/109019818401100101

[22] Equestrian Canada . COVID‐19 return to business operations framework. [cited 2023 April 1]. Available from: https://www.equestrian.ca/cdn/storage/resources_v2/cRSrWCZPbutzEN8Qg/original/cRSrWCZPbutzEN8Qg.pdf

[23] Malterud K , Siersma VD , Guassora AD . Sample size in qualitative interview studies: guided by information power. Qual Health Res. 2016;26(13):1753–1760. 26613970 10.1177/1049732315617444

[24] Braun V , Clarke V . In: Maher A , editor. Thematic analysis: a practical guide. London: SAGE Publications Inc; 2022.

[25] De Carlo M . Paradigms, theories, and how they shape a researcher’ approach. Scientific enquiry in social work; Roanoke: Open Social Work Education; 2018.

[26] Ontario Ministry of Agriculture Food and Rural Affairs . Guidelines for the vaccination of horses. [cited 2023 April 30]. Available from: http://omafra.gov.on.ca/english/livestock/horses/facts/info_vaccine.htm#:~:text=The%20basic%20or%20core%20vaccines,tetanus%20and%20West%20Nile%20virus

[27] Reason J . Understanding adverse events: human factors. Qual Health Care. 1995;4(2):80–89. 10151618 10.1136/qshc.4.2.80 PMC1055294

[28] D'Amore R . What is the ‘Swiss cheese model’ and how can it apply to coronavirus? Global News. 2020; [cited 2023 April 30]. Available from: https://globalnews.ca/news/7393839/coronavirus-swiss-cheese-model/

[29] Seshia SS , Bryan Young G , Makhinson M , Smith PA , Stobart K , Croskerry P . Gating the holes in the Swiss cheese (part I): expanding professor Reason's model for patient safety. J Eval Clin Pract. 2018;24(1):187–197. 29168290 10.1111/jep.12847 PMC5901035

[30] World Health Organization . Pandemic fatigue: reinvigorating the public to prevent COVID‐19. 2020 Available from: http://apps.who.int/bookorders

[31] Spence KL , Rosanowski SM , Slater J , Cardwell JM . Challenges to exotic disease preparedness in Great Britain: the frontline veterinarian's perspective. Equine Vet J. 2022;54(3):563–573. 34043828 10.1111/evj.13469

[32] Nixon J . Learning about equine biosecurity. Vet Rec. 2015;176(23):i–ii. 10.1136/vr.h2981 26044699

[33] Anderson LR , Mellor JM . Predicting health behaviors with an experimental measure of risk preference. J Health Econ. 2008;27(5):1260–1274. 18621427 10.1016/j.jhealeco.2008.05.011

[34] Satoh M , Sato N . Relationship of attitudes toward uncertainty and preventive health behaviors with breast cancer screening participation. BMC Womens Health. 2021;21(1):171. 33882923 10.1186/s12905-021-01317-1 PMC8061057

[35] Manyweathers J , Field H , Longnecker N , Agho K , Smith C , Taylor M . “Why won't they just vaccinate” horse owner risk perception and uptake of the Hendra virus vaccine. BMC Vet Res. 2017;13(1):103. 28407738 10.1186/s12917-017-1006-7 PMC5390447

[36] Douglas M , Wildavsky A . Risk and culture: an essay on the selection of technological and environmental dangers. Berkeley: University of California Press; 1983.

[37] Ferrari M . Risk perception, culture, and legal change: a comparative study on food safety in the wake of the mad cow crisis. London: Routledge; 2009.

[38] Barley JM , Latanfi B . Bystander intervention in emergencies: diffusion of responsibility. J Pers Soc Psychol. 1968;8(4, Pt 1):377–383. 5645600 10.1037/h0025589

[39] Hussain I , Shu R , Tangirala S , Ekkirala S . The voice bystander effect: how information redundancy inhibits employee voice. Acad Manage J. 2019;62(3):828–849.

[40] Scheufele DA , Moy P . Twenty‐five years of the spiral of silence: a conceptual review and empirical outlook. Int J Public Opin Res. 2000;12(1):3–28.

[41] Voigt M , Russell M , Hiney K , Richardson J , Borron A , Brady C . Show horse welfare: evaluating stock‐type show horse industry legitimacy. J Agric Environ Ethics. 2015;28(4):647–666.

[42] Horseman SV , Buller H , Mullan S , Whay HR . Current welfare problems facing horses in Great Britain as identified by equine stakeholders. PLoS One. 2016;11(8):e0160269. 27501387 10.1371/journal.pone.0160269 PMC4976980

[43] National Farm Animal Care Council . Code of practice for the care and handling of equines. 2013.

[44] Shepperd J , Malone W , Sweeny K . Exploring causes of the self‐serving bias. Soc Personal Psychol Compass. 2008;2(2):895–908.

[45] Xun Z , Gilman S . Placing the blame: what if “they” REALLY are responsible? J Med Humanit. 2021;42(1):17–49. 33738707 10.1007/s10912-020-09674-y PMC7972006

[46] Dariotis JK , Sloane SM , Smith RL . “I took it off most of the time ‘cause I felt comfortable’”: unmasking, trusted others, and lessons learned from a coronavirus disease 2019 reinfection: a case report. J Med Case Reports. 2021;15(1):557. 10.1186/s13256-021-03033-8 PMC8581599 34763726

[47] Sauer MA , Truelove S , Gerste AK , Limaye RJ . A failure to communicate? How public messaging has strained the COVID‐19 response in the United States. Health Secur. 2021;19:65–74. 33606575 10.1089/hs.2020.0190 PMC9195491

[48] Krugman P . A plague of wilful ignorance. The New York Times. 2020 [cited 2023 May 7]. Available from: https://www.nytimes.com/2020/06/22/opinion/coronavirus-trump.html

[49] Deem SL , Lane‐de Graaaf KE , Rayhel EA . Introduction to One Health: an interdisciplinary approach to planetary health. Hoboken, NJ: John Wiley & Sons, Incorporated; 2019.

[50] Institute for Healthcare Improvement . How to reduce implicit bias. 2017 [cited 2023 May 7]. Available from: https://www.ihi.org/communities/blogs/how‐to‐reduce‐implicit‐bias#:~:text=Strategies%20to%20Reduce%20Implicit%20Bias&text=Counter%2Dstereotypic%20imaging%20%E2%80%94%20Imagining%20the,doctor's%20office%20or%20health%20center

