# Supplementary material for: Biosecurity perceptions among Ontario horse owners during the COVID‐19 pandemic
Source: Equine Vet J. 2024 Jun 27;57(2):459–70. doi: 10.1111/evj.14115 (PMC11807931; doi:10.1111/evj.14115)
Supplement: Supplementary file 1 — Data S1: Supporting information. [file EVJ-57-459-s002.docx]

**WAHRNEHMUNG DER BIOLOGISCHEN SICHERHEIT UNTER PFERDEBESITZERN IN ONTARIO WÄHREND DER COVID-19-PANDEMIE**

Juliet A. Germann*, Terri L. O’Sullivan, Amy L. Greer and Kelsey L. Spence

Department of Population Medicine, Ontario Veterinary College, University of Guelph

*Corresponding author: Juliet A. Germann, germannj@uoguelph.ca,

1.1 SUMMARY

**Hintergrund:** Krankheitsausbrüche stellen eine große Herausforderung für die Gesundheit und das Wohlergehen von Pferden sowie für die wirtschaftliche Stabilität der internationalen Pferdewirtschaft dar. Dies ist ein besonderes Problem in Ontario, Kanada, wo es häufig zu Ausbrüchen von Infektionskrankheiten der Atemwege bei Pferden gekommen ist. Trotz dieser Risiken gibt es nur wenige Untersuchungen darüber, ob Pferdebesitzer in Ontario ausreichende Biosicherheitsmaßnahmen ergreifen, um das Risiko von Pferdekrankheiten zu mindern, und ob aktuelle Ereignisse wie die COVID-19-Pandemie die Einstellung zu Biosicherheitspraktiken bei Pferden beeinflussen.

**Ziele:** Untersuchung der Wahrnehmungen, Einstellungen und Erfahrungen von Pferdebesitzern in Ontario in Bezug auf die Biosicherheit in landwirtschaftlichen Betrieben während der COVID-19-Pandemie.

**Studiendesign:** Qualitative Studie mit virtuellen halbstrukturierten Interviews.

**Methoden:** Die Teilnehmer (Pferdebesitzer, regelmäßige Reiter und Teilpächter) wurden über soziale Medien rekrutiert, in denen Anzeigen von Pferde- und Tierarztorganisationen geteilt wurden. Die Interviews wurden zwischen Juni und September 2022 virtuell durchgeführt und mit Hilfe einer reflexiven thematischen Analyse ausgewertet.

**Ergebnisse:** Unter den 14 Teilnehmern wurden drei Hauptthemen in Bezug auf die Wahrnehmung der Biosicherheit ermittelt. Die Teilnehmer verließen sich auf minimale Präventivmaßnahmen (z. B. Impfungen), wenn das Risiko einer Erkrankung als gering eingeschätzt wurde, führten jedoch zusätzliche Maßnahmen wie Quarantäne und Händewaschen ein, wenn das Risiko einer Erkrankung als hoch eingeschätzt wurde. Die von den Teilnehmern gewählten Biosicherheitspraktiken entsprachen häufig den Empfehlungen des Stallbetreibers. Darüber hinaus waren die Teilnehmer der Ansicht, dass die Verantwortung für die Biosicherheit nicht gleichmäßig auf alle Pferdebesitzer verteilt ist, wobei der Schwerpunkt eher auf denjenigen liegt, die in Situationen mit hohem Risiko für die Verbreitung von Krankheiten involviert sind. Trotz der Erfahrungen mit der Biosicherheit während der COVID-19-Pandemie wandten die Pferdebesitzer diese Praktiken bei der Pflege ihrer Pferde nicht konsequent an.

**Hauptlimitationen:** Die hier berichteten Perspektiven stammen von einer kleinen Stichprobe von Pferdebesitzern und sind möglicherweise nicht auf alle Bevölkerungsgruppen übertragbar.

**Schlussfolgerungen:** Unsere Ergebnisse zeigen, dass Pferdebesitzer einen besseren Zugang zu und eine stärkere Beteiligung an Aufklärungsinitiativen benötigen, die die Bedeutung und den Zweck aller Biosicherheitsmaßnahmen hervorheben.
