# Supplementary material for: Biosecurity perceptions among Ontario horse owners during the COVID‐19 pandemic
Source: Equine Vet J. 2024 Jun 27;57(2):459–70. doi: 10.1111/evj.14115 (PMC11807931; doi:10.1111/evj.14115)
Supplement: Supplementary file 2 — File S1: Interview guide. [file EVJ-57-459-s001.pdf]

Equine Veterinary Journal BEVA

File S1

Interview Guide

How did you first get involved with horses?

- How long have you been involved with horses?

How many horses do you have?

Tell me about the horse(s) that you have now?

- What kinds of activities (what type of riding? Competition, leisure, etc.)
- Are you the sole owner of the horse? → Who is the owner?
    - Do you have a part-boarder? Are you a part-boarder?

Do you keep your horse at home, or do you board them at a stable?

- How many horses are at your stables/facilities/location?

Tell me about the other horses and horse owners at your facility:

- Are they recreational and/or competition? What age group? (Teenagers, adults, etc.)
- Could you describe the community at your barn/stable? (Close knit, distant, etc.)

What are your current barn management arrangements?

- Who's your barn manager and/or owner?
    - What's your relationship like with them?
    - Who is your go-to person when you have questions or concerns?

What is a normal day of caring for your horse?

- Who cares for your horse daily?
- Thinking back to March 2020 when the pandemic first started, how did the normal operations at the barn change?

("Now you know this project is about infectious diseases and biosecurity…")

If someone were to ask you what biosecurity means, what would you say?

Do you use the term biosecurity at the barn?

- How often is it brought up in conversation?
- What kinds of things do you talk about?
- IF they say "we never talk about it": Why do you think the term isn't used often?

If you were going to look up some information on biosecurity, where would you go?

- Why there?
- What would you consider to be a reliable source of information and why?

What sorts of management practices do you think of when you hear the word biosecurity?

- Typical biosecurity measures include vaccinations, sanitation, quarantines, and so on. Do you use any of these practices?

Option A (Horse is stabled at home facility):

What sorts of biosecurity practices are at your facility?

- Do you consider these to be useful or effective?

What are you most concerned about when you think about contagious diseases at your facilities?

- (If diseases mentioned): Why those diseases?

Are contagious equine diseases something you're concerned about when you leave the facility?

- If yes: what are your concerns?
  - Why do you think you have these concerns?
- If no: why not?
  - At what point do you think you would be concerned?

Have you had any experience with equine infectious disease during the pandemic?

- How about before the pandemic? (What changed? What would you do differently?)

Do other horses ever come board at your facility?

- If yes: What is the process of boarding (is there a quarantine process?), are vaccines checked? (Which ones are required/preferred)
- What is the common length of time for a horse to board?

Have you ever boarded your horse at another facility?

- What was your experience with that?
- Is there anything biosecurity related that you would have changed during your time boarding?

In general, is there something other horse facilities should or should not be doing with regards to biosecurity?

  - How do you feel about how other horse owners are applying biosecurity protocols?

What challenges exist in trying to use biosecurity measures at your barn? Such as sanitation, quarantining, or vaccination?

- If not: what challenges could you anticipate happening if a new biosecurity measure had to be implemented (i.e., quarantine policy?)

Since the start of the pandemic, have you seen an increase or decrease in biosecurity implementation among horse owners or horse facilities? How so?

If you wanted to see any changes about biosecurity at your barn, what would they be? What about in the wider horse industry?

- ("everything is working well…"): Can you talk a little bit about what you think is working well?

Is there anything else you would like to add?

Option B (Horse is stabled at a boarding stable):

Does your barn have a policy around biosecurity?

- "YES": Tell me about it?
    - Who makes the decisions at the stables to implement/determine these protocols?
    - Do you consider these to be useful or effective?
- "NO": What do you think about that?

What are you most concerned about when you think about equine contagious diseases at your facility?

- (If diseases mentioned): Why those diseases?

Are contagious equine diseases something you're concerned about when you leave the facility?

- If yes: what are your concerns?
    - Why do you think you have these concerns?
- If no: why not?
    - At what point do you think you would be concerned?

Have you had any experience with equine infectious disease during the pandemic?

- How about before the pandemic?

How do you feel about the level of compliance with the policies that are in place? Are horse owners upholding the protocols implemented?

- Are people generally happy and willing to participate?

In general, is there something other horse facilities should or should not be doing with regards to biosecurity?

- How do you feel about how other horse owners think of or are applying biosecurity protocols?

What challenges exist in trying to use biosecurity measures at your barn? Such as sanitation, quarantining, or vaccination?

- If not: what challenges could you anticipate happening if a new biosecurity measure had to be implemented (i.e., quarantine policy?)

If you wanted to see any changes about biosecurity at your barn, what would they be? What about in the wider horse industry?

- ("everything is working well…"): Can you talk a little bit about what you think is working well?

Is there anything else you would like to add?
